# The multistep hypothesis of ALS revisited: The role of genetic mutations

**DOI:** 10.1212/WNL.0000000000005996

**Published:** 2018-08-14

**Authors:** Adriano Chiò, Letizia Mazzini, Sandra D'Alfonso, Lucia Corrado, Antonio Canosa, Cristina Moglia, Umberto Manera, Enrica Bersano, Maura Brunetti, Marco Barberis, Jan H. Veldink, Leonard H. van den Berg, Neil Pearce, William Sproviero, Russell McLaughlin, Alice Vajda, Orla Hardiman, James Rooney, Gabriele Mora, Andrea Calvo, Ammar Al-Chalabi

**Affiliations:** From the “Rita Levi Montalcini” Department of Neuroscience (A. Chiò, A. Canosa, C.M., U.M., M.B., M.B., A. Calvo), University of Torino; Institute of Cognitive Sciences and Technologies (A. Chiò), National Research Council, Rome; ALS Center (L.M., E.B.), Department of Neurology, Azienda Ospedaliera Universitaria Maggiore della Carità; Department of Health Sciences (S.D., L.C.), Interdisciplinary Research Center of Autoimmune Diseases, “Amedeo Avogadro” University of Eastern Piedmont, Novara, Italy; Department of Medical Statistics (N.P.), London School of Hygiene and Tropical Medicine, UK; Centre for Public Health Research (N.P.), Massey University Wellington Campus, New Zealand; Department of Neurology and Neurosurgery (J.H.V., L.H.v.d.B.), Brain Center Rudolf Magnus, University Medical Center Utrecht, the Netherlands; Academic Unit of Neurology (R.M., A.V., O.H., J.R.), Trinity Biomedical Sciences Institute, Trinity College Dublin, Ireland; Istituti Clinici Scientifici Maugeri (G.M.), IRCCS Milano, Italy (Gabriele Mora); and King's College London (W.S., A.A.-C.), Institute of Psychiatry, Psychology and Neuroscience, Maurice Wohl Clinical Neuroscience Institute, UK.

## Abstract

**Objective:**

Amyotrophic lateral sclerosis (ALS) incidence rates are consistent with the hypothesis that ALS is a multistep process. We tested the hypothesis that carrying a large effect mutation might account for ≥1 steps through the effect of the mutation, thus leaving fewer remaining steps before ALS begins.

**Methods:**

We generated incidence data from an ALS population register in Italy (2007–2015) for which genetic analysis for *C9orf72, SOD1, TARDBP*, and *FUS* genes was performed in 82% of incident cases. As confirmation, we used data from ALS cases diagnosed in the Republic of Ireland (2006–2014). We regressed the log of age-specific incidence against the log of age with least-squares regression for the subpopulation carrying disease-associated variation in each separate gene.

**Results:**

Of the 1,077 genetically tested cases, 74 (6.9%) carried *C9orf72* mutations, 20 (1.9%) had *SOD1* mutations, 15 (1.4%) had *TARDBP* mutations, and 3 (0.3%) carried *FUS* mutations. In the whole population, there was a linear relationship between log incidence and log age (*r*^2^ = 0.98) with a slope estimate of 4.65 (4.37–4.95), consistent with a 6-step process. The analysis for *C9orf72*-mutated patients confirmed a linear relationship (*r*^2^ = 0.94) with a slope estimate of 2.22 (1.74–2.29), suggesting a 3-step process. This estimate was confirmed by data from the Irish ALS register. The slope estimate was consistent with a 2-step process for *SOD1* and with a 4-step process for *TARDBP*.

**Conclusion:**

The identification of a reduced number of steps in patients with ALS with genetic mutations compared to those without mutations supports the idea of ALS as a multistep process and is an important advance for dissecting the pathogenic process in ALS.

Amyotrophic lateral sclerosis (ALS) is a neurodegenerative disorder characterized by a progressive loss of cortical, bulbar, and spinal motor neurons, often associated with an involvement of prefrontal cortex. There are indications that the degenerative process in ALS is the consequence of a combination of genetic and environmental factors. More than 20 genes have been detected as causes of ALS.^1^ Several environmental factors have been proposed, but none of them, with the possible exception of cigarette smoking and military service, are consistently associated with ALS.^2–5^ About 20% of ALS heritability is attributable to common genetic variation compared with an overall heritability of 60% in studies based on concordance of monozygotic twin pairs.^6–8^

In a previous study, we used the Armitage-Doll model derived from cancer research to assess whether ALS incidence is consistent with a multistep process and, if so, to estimate the number of steps (n) required for ALS to develop.^9,10^ The model can be briefly conceptualized as follows: if we assume that ALS is caused in a single-step molecular process, then the incidence in a particular year will be proportional to the risk of undergoing the step, which in turn depends on exposure to the relevant disease-causing factor. The probability a second molecular step has occurred by that year is dependent on the risk of exposure to the relevant factor per year and the number of years of exposure, or age, and this is true for any subsequent step. Thus, incidence is proportional to the product of the risks of undergoing the first step and the subsequent steps. This concept implies a logarithmic increase in incidence with age, obeying a power law in which 1 less than the number of steps, n − 1, relates to the rate of increase. As a result, taking logs of the age at onset and incidence rates has the form of a straight-line equation with slope n − 1 if a multistep model applies. Our previous study found a linear relationship with a slope estimate of 5, indicating that the process leading to ALS needs on average 6 steps.^9^ Considering the large heterogeneity of ALS in terms of clinical presentation, progression, and outcome, it is likely that the number of steps varies in specific subgroups of patients. For example, those carrying a large effect mutation might have ≥1 steps accounted for by the effect of the mutation and thus have fewer remaining steps before ALS is established. We therefore tested this hypothesis using the Armitage-Doll model in genetically defined patient subgroups from a population-based cohort.

## Methods

All people with ALS diagnosed in Piemonte and Valle d'Aosta, Italy, in the period of 2007 to 2015 were eligible to be enrolled in the study. Cases were identified through the Piemonte and Valle d'Aosta Register for ALS (PARALS). PARALS is a prospective epidemiologic register based on the collaboration of the neurologic departments of the 2 Italian regions. ALS cases are ascertained through several concurrent sources (hospital admission, etc.). ALS diagnosis is based on El Escorial revised criteria. Cases with definite, probable, and probable laboratory-supported El Escorial diagnosis during the course of the disease are included in the register. A detailed description of register methodology is reported elsewhere.^11^ The cohort included in this study is different from that included in the previous one, which was based on patients incident in the 1995–2004 period.

As a confirmation cohort, we used the data from ALS cases diagnosed in the Republic of Ireland in the 2006–2014 period. ALS cases were identified though the Irish ALS register.^12^ Although similar cohorts exist for the other registers studied in our original report, the genetic data either are not complete enough or do not overlap enough with the population data to allow similar analysis.

### Genetic analysis

All cases were tested for mutations in *SOD1* (all exons), *TARDBP* (exon 6), *FUS* (exons 14 and 15), and *C9orf72* with the use of standard methodology described elsewhere.^13^ *C9orf72* repeat length was determined with repeat primed PCR. Normal was defined as ≤28 repeats.

### Statistical analysis

The Armitage-Doll methodology was used,^10^ under the same assumptions as our previous report.^9^ In brief, a plot of the log of ALS incidence against log age will be linear if a multistep model applies and will have slope n − 1, i.e., 1 less than the number of steps needed for disease onset. According to the pattern identified in cancer, the model predicts that the slope will be approximately linear but will decrease (and therefore will be less than linear) at older age groups due to a substantial proportion of the population having undergone 1 or more of the earlier steps.

Following this model, we calculated the incidence rates per 100,000 person-years in 5-year age groups for people 35 to 74 years of age. We excluded the youngest age groups (those <35 years) because of the small number of patients and the older age groups (those >74 years) because of the risk of underascertainment or cohort effect; this reflects also the finding in some cancers for which the log incidence and log age association is nonlinear in the older age groups.^10^

We then performed a preliminary analysis of the log incidence against log age on all cases (i.e., both mutated and nonmutated) to verify whether our population followed a multistep model and to replicate our previous findings. Second, we assessed separately patients with familial and those with nonfamilial ALS. Third, we assessed the incidence of ALS for cases involving each single gene. To correctly calculate incidence, the population used for the denominator should correspond to the population used for the numerator. For example, for ALS incidence in those carrying a *C9orf72* mutation, the correct denominator to use would be the count of all people in the population carrying a *C9orf72* mutation. This information was available only for the cases but not for the general population. However, because the relevant mutations do not in general markedly increase mortality apart from their effects on ALS, we assumed that the proportions of the population carrying a specific mutation would not differ substantially by age group (e.g., the proportions with the *C9orf72* mutation would be similar in the 40- to 44- and 60- to 64-year age groups). Under this assumption, it is then reasonable to use the total population as the denominators in the analyses for specific genes (e.g., cases involving the *C9orf72* mutation) because this would involve multiplying the relevant age-specific population denominator by an unknown but fixed constant (e.g., if 5% of the population carry a particular mutation, then the total population denominator would be 20 times that of the unknown population subgroup carrying this mutation). Thus, all of the age-specific incidence rates would be overestimated by an unknown but fixed multiplying factor; this in turn would affect the age-specific incidence rates but would have no effect on the slope of the graph of log incidence against log age.

### Standard protocol approvals, registrations, and patient consents

The Piedmont regional government has recognized the Piemonte ALS Registry as a Registry of High Sanitary Interest (regional law, April 11, 2012, No. 4). Accordingly, PARALS has the right to access all the existing databases owned by the regional administration and to obtain clinical information about patients with ALS from public and private hospitals and general practitioners. The study was approved by the ethics committee of the Città della Salute e della Scienza of Turin. The register database is anonymized and treated according to Italian Data Protection Code. Patients sign a written informed consent. The Irish ALS Register complies with Irish Data protection legislation (1988 and 2003) and has been approved by the Beaumont Hospital Ethics Committee (02/28 and 05/49).

### Data availability

Anonymized data will be shared by request from any qualified investigator.

## Results

Of the 1,309 cases incident during the 2007–2015 period, 1,077 (82.2%) underwent genetic analysis of all 4 genes, 93.5% (1,030) of those followed up by the 2 ALS multidisciplinary centers and 21.7% (47) of those followed up by general neurology departments. Patients who did not undergo genetic analysis were older and more frequently had a bulbar onset than those who were tested (table 1). *C9orf72* mutations were detected in 74 cases (6.9%), *SOD1* in 20 (1.9%), *TARDBP* in 15 (1.4%), and *FUS* in 3 (0.3%). One patient carried both a *C9orf72* expansion and the p.Asn390Ser heterozygous missense mutation of the *TARDBP* gene. A list of *SOD1, TARDBP*, and *FUS* mutations is reported in table 2.

**Table 1:**
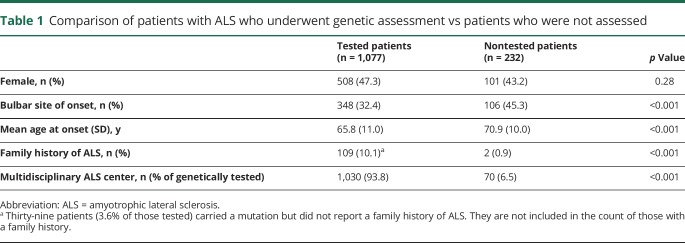
Comparison of patients with ALS who underwent genetic assessment vs patients who were not assessed

**Table 2:**
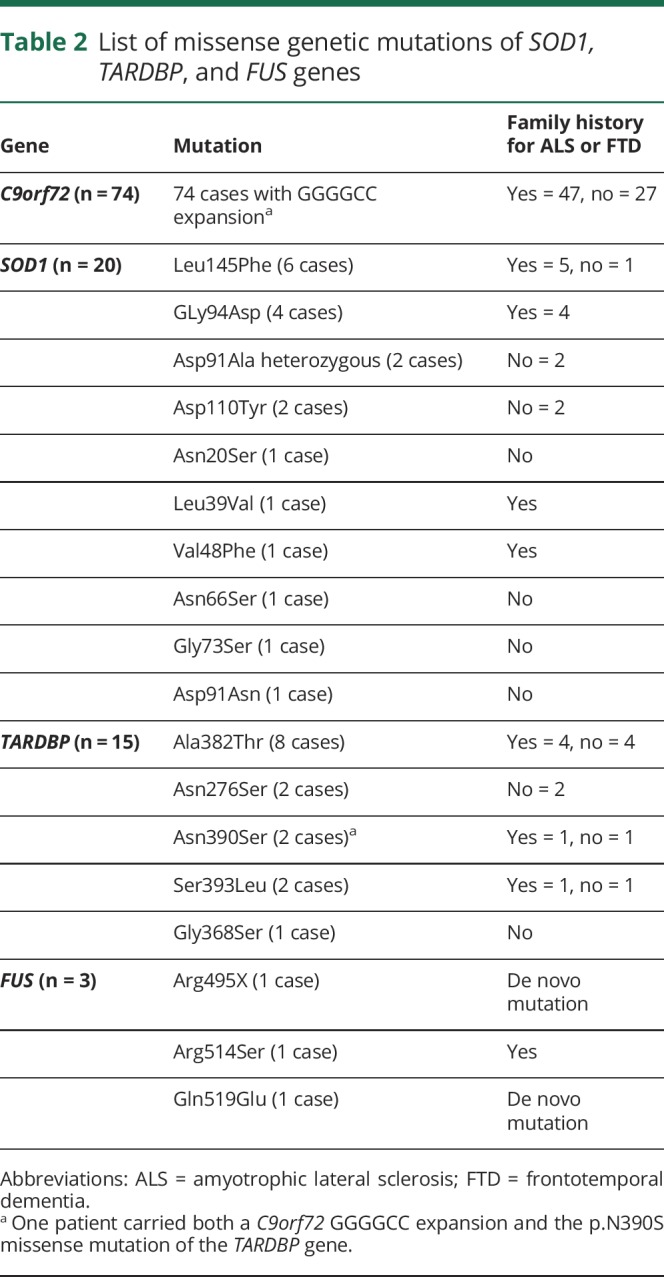
List of missense genetic mutations of *SOD1, TARDBP*, and *FUS* genes

**Figure 1:**
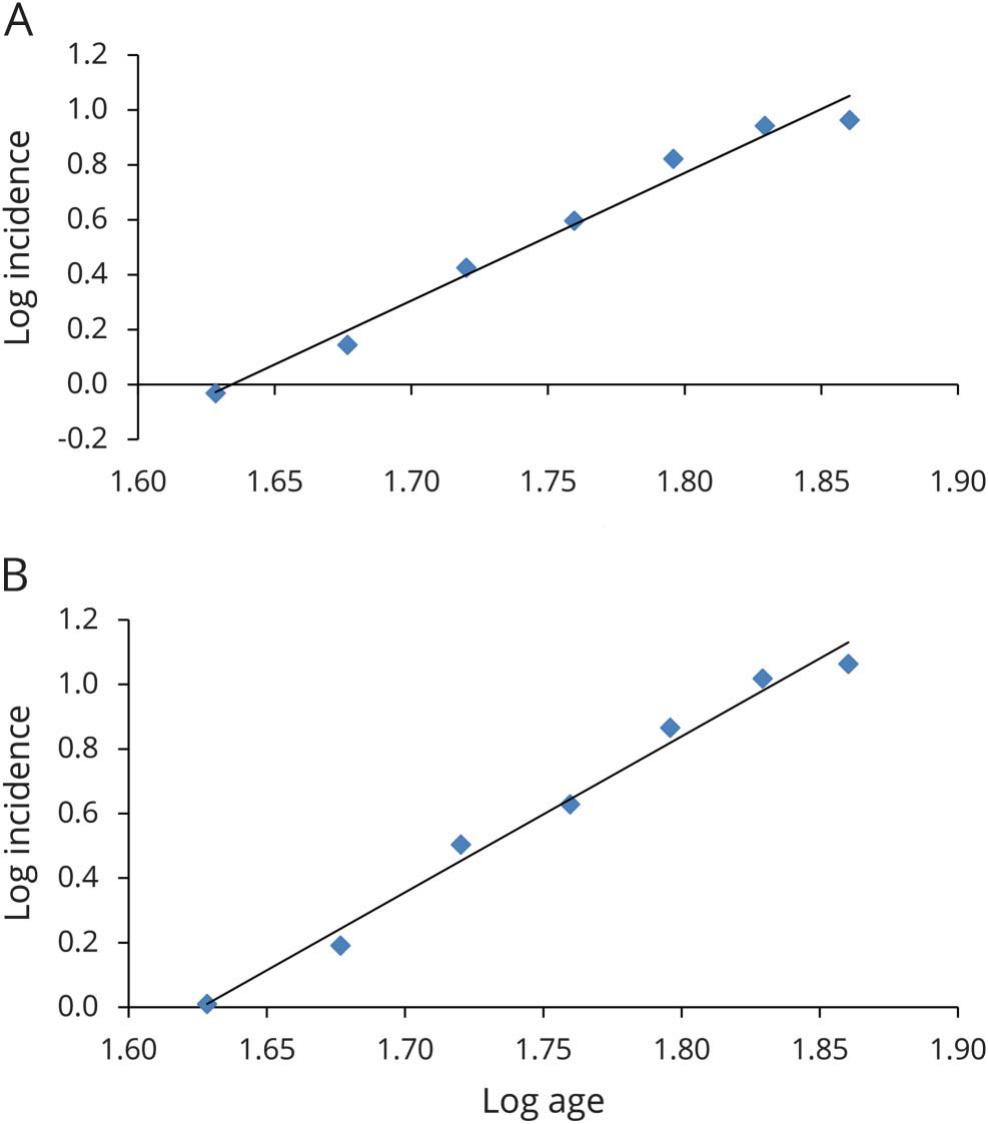
Slope estimation for all patients with ALS (A) Log incidence vs log age for all patients with incident amyotrophic lateral sclerosis (ALS) who have been genetically tested (n = 1,077) (y = 4.65x − 7.60, *r*^2^ = 0.98). (B) Log incidence vs log age for all patients with incident ALS in the register (n = 1,309) (y = 4.83x − 7.85, *r*^2^ = 0.99).

In the 1,077 patients with genetic test data, there was a linear relationship between log incidence and log age (*r*^2^ = 0.98) with a slope estimate of 4.65 (95% confidence interval [CI] 4.37–4.95), consistent with a 6-step process (figure 1A), replicating our previous findings. A similar result (*r*^2^ = 0.99) was obtained when all 1,309 incident cases (figure 1B) were included. There was no effect of sex (data not shown).

**Figure 2:**
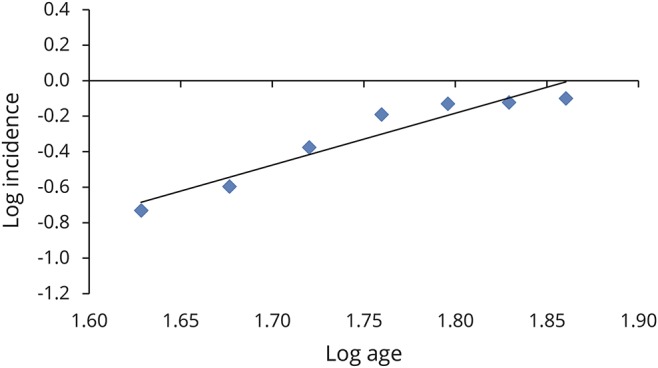
Slope estimation for all those with familial ALS Log incidence vs log age for all patients with incident familial amyotrophic lateral sclerosis (ALS) (n = 111) (y = 2.95x − 5.45, *r*^2^ = 0.92).

When the 109 patients with definite or probable familial ALS (10.1% of the total) were considered,^14^ there was a linear relationship between log incidence and log age, with a slope estimate of 2.95 (95% CI 2.43–3.57), consistent with a 4-step process (figure 2).

**Figure 3:**
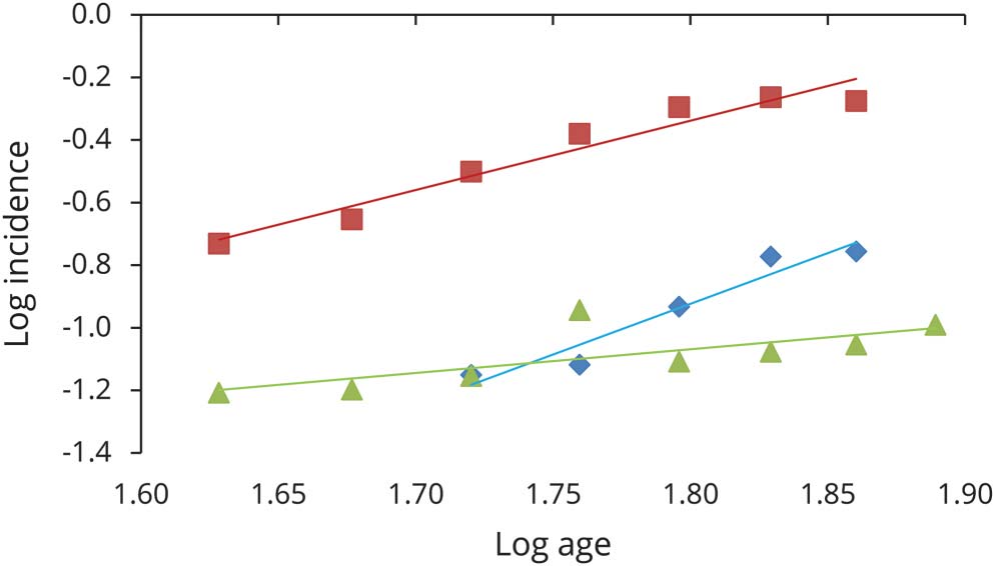
Slope estimation for all patients with ALS carrying a mutation in 1 of 4 tested genes Log incidence vs log age for *C9orf72* amyotrophic lateral sclerosis (ALS) (74 cases) (y = 2.22x − 4.33, *r*^2^ = 0.94) (red line), for *SOD1* ALS (20 cases) (y = 0.758x − 2.43, *r*^2^ = 0.53) (green line), and for *TARDBP* ALS (15 cases) (y = 3.24x − 6.76, *r*^2^ = 0.93) (blue line). The fit to a straight line is good, consistent with a multistep model.

The analysis for *C9orf72*-mutated patients confirmed a linear relationship (*r*^2^ = 0.94) with a slope estimate of 2.22 (95% CI 1.74–2.79), suggesting a 3-step process (figure 3A). Similarly, a linear relationship was found for *SOD1*-mutated patients (*r*^2^ = 0.53, n − 1 = 0.76, 95% CI 0.46–1.17), consistent with a 2-step process (figure 3B), and for *TARDBP* (*r*^2^ = 0.93, n − 1 = 3.24, 95% CI 2.21–4.13), consistent with a 4-step process (figure 3C). Because of the very small number of cases carrying *FUS* mutations, we did not estimate the slope. When we considered the 45 patients with familial ALS who were negative for the 4 tested genes, the linear relationship was confirmed (*r*^2^ = 0.95, n − 1 = 3.71, 95% CI 2.67–4.53), consistent with a 5-step process (figure not shown). These data are summarized in table 3.

**Table 3:**
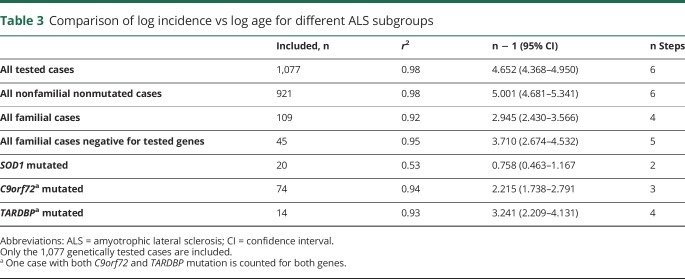
Comparison of log incidence vs log age for different ALS subgroups

We next analyzed patients from the Irish ALS register. The register includes 597 genetically tested patients (56.3% of incident patients in the 2006–2014 period), of whom 67 carried a *C9orf72* expansion. In the 597 patients with genetic test data, there was a linear relationship between log incidence and log age (*r*^2^ = 0.93) with a slope estimate of 5.09 (95% CI 4.69–5.52), consistent with a 6-step process. In the *C9orf72*-expanded cases, results were similar to those of the Piemonte register (*r*^2^ = 0.66, slope estimate 2.47, 95% CI 1.91–3.13, consistent with a 3-step process). Finally, the 530 Irish patients without a *C9orf72* expansion had a slope estimate of 5.35 (95% CI 4.92–5.82) (*r*^2^ = 0.95). No patients with *SOD1* mutations and only 2 with *TARDBP* missense mutations were identified in the Irish ALS register, making it impossible to assess the effects of these genes.

**Figure 4:**
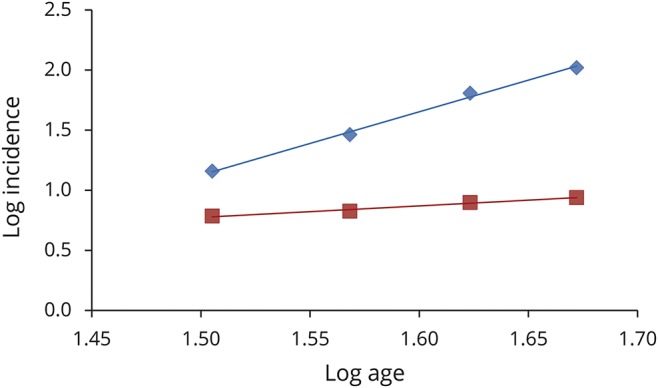
Slope estimation for patients with type 1 and 2 diabetes mellitus Data from Piemonte diabetes register.^14^ Log incidence vs log age for type 1 diabetes mellitus (y = 0.96x − 0.66, *r*^2^ = 0.98) (red line) and type 2 diabetes mellitus (y = 5.28x − 6.78, *r*^2^ = 1.0) (blue line).

For comparison, we assessed the slope for type 1 and 2 diabetes mellitus using data from the Piemonte register for diabetes mellitus for the 30- to 49-year age groups (figure 4).^15^ In keeping with our findings on ALS, the slope estimate for type 1 diabetes mellitus, a highly genetically determined disease, was 0.96 (95% CI 0.62–1.13) (*r*^2^ = 1.0), consistent with a 2-step process, while that of type 2 diabetes mellitus, a multifactorial disease with a polygenic architecture, was 5.27 (95% CI 4.50–6.18) (*r*^2^ = 0.98), consistent with a 6-step process.

## Discussion

We have found that in patients carrying a genetic mutation, the slope of the graph of log incidence and log age is lower than that of cases who do not carry these mutations. This in turn implies that the number of steps necessary to start the neurodegenerative process in genetically mediated ALS is reduced compared to cases without mutation. The number of steps varies according to the mutated gene and is lower for *SOD1* (2), intermediate for *C9orf72* (3), and higher for *TARDBP* (4). The number of steps identified in patients without mutation is 6, consistent with our previous report.^9^ In particular, the slope in the patients from the Piemonte register reported in that article, which was based on incident cases in the 1995–2004 period, is almost identical to that found in the present report, which was based on the incident cases in the 2007–2015 period. Furthermore, the slope for *C9orf72,* as well as the overall slope of genetically tested patients, was confirmed in the Irish ALS population. These findings suggest that a genetic lesion alone might account for up to 4 molecular steps, leaving only 2 further, likely environmental, steps for those with *SOD1* mutation, for example. This argues for the concentration of efforts in dissecting environmental risk factors in individuals with identified mutations rather than those with apparently sporadic ALS, because such environmental factors will be fewer in number per person and likely of larger effect size as a result.

It is generally recognized that ALS is a multifactorial disease, characterized by interplay between genetic and environmental factors. Although several ALS-related genes are known, it is increasingly clear that genetic mutations alone cannot fully explain the pathologic process in ALS but rather that genes can be considered triggers of the degenerative process. A similar role can be attributed to environmental toxins. However, we have very little information about the possible exogenous factors involved in ALS. Cigarette smoking may be a risk factor in ALS^5,16^; other suggested factors are physical activity, participating in professional sports, and physical trauma.^4^ Protective factors have also been hypothesized such as diabetes mellitus^17^ and an unfavorable lipid profile.^18,19^ All these factors could act on the genome through epigenomic interactions. For example, smoking induces DNA hypermethylation in specific CpG sites, which persists for years after cessation of smoking,^20^ or may induce somatic nucleic acid changes.^5,21^ It is likely that the remaining steps in different genetic subgroups may originate from 1 or more of these risk factors.

Besides our current results, more observations fit the multistep hypothesis in ALS. First, there are indications that ALS can be an oligogenic disease. In fact, there are several reports of patients carrying ≥2 mutations of different ALS-related genes.^22^ In a study of 391 patients with ALS that assessed variants in 17 genes, 3.8% had variants in >1 gene.^23^ In that series, the burden of rare variants in known ALS genes significantly reduced the age at onset of symptoms.^23,24^ In the present series, 1 patient had both a *C9orf72* expansion and a heterozygous mutation of the *TARDBP* gene, even though we assessed only 4 genes. Second, besides “causative” genes, several other genes have been reported to modify ALS phenotype such as *UNC13A*, *ATXN2*, and *CAMTA1*,^25–27^ suggesting that variants in these genes modify the sequential process, either accelerating or slowing it.

Nongenetic elements such as environmental factors^28^ and aging also likely trigger molecular steps. However, consistent with other reports, the slope is the same between sexes in all analyses, suggesting that there is no effect of sex on the assumed cascade.^29^

There appears to be some relationship between the number of remaining steps identified for each mutated gene and the penetrance of mutation in the gene. Such a relationship is consistent with a multistep model because a greater number of remaining steps will correspond to a lower probability of exposure to all the steps and therefore reduce the probability of disease given a specific genotype. For example, *C9orf72* expansion mutation penetrance has been estimated to be 60% at the age of 60 years and 91% at the age of 80 years^30,31^ and corresponds to 3 remaining steps. At least 3 mechanisms might regulate *C9orf72* penetrance: the size of the GGGGCC expansion, DNA methylation and transcriptional downregulation of the promoter,^32^ and the presence of additional mutations.^33^

The penetrance of *TARDBP* mutation is much lower than that of *C9orf72* (60% at 80 years for the p.Ala382Thr mutation),^34^ and it leaves 4 remaining steps, more than for the other 2 genes. The lowest number of remaining steps, 2, has been estimated for *SOD1* mutation. However, *SOD1* penetrance varies across the different mutations. For example, in a study on pedigrees dating back to the 18th century, carriers of p.Glu101Gly, p.Ile114Thr, and p.Val149Gly *SOD1* mutations were reported to have a penetrance of >95% at the age of 78 years.^35^ Similarly, the penetrance of the p.Ala5Val mutation, the commonest in the United States, has been estimated to be 91% at the age of 80 years.^36^ Other mutations have a much reduced penetrance; an example is the p.Asp91Ala mutation, which is transmitted with a recessive inheritance in people of Scandinavian ancestry and with a dominant inheritance, albeit with a low penetrance, in the other populations.^37,38^ Most of the *SOD1* mutations we identified are regarded as having very high penetrance and would therefore be expected to account for more steps than low penetrance mutations.

This study has some weaknesses. First, it was not possible to genotype all patients with incident ALS. Nontested patients were older and more frequently had bulbar onset than those who were tested. However, we could obtain DNA for >80% of incident patients, a high proportion in an epidemiologic setting. Second, only the 4 more commonly mutated ALS genes were assessed. However, nontested genes account for only a fraction of patients with ALS in European-derived populations. Third, the estimation of the slope was performed on the relatively small number of genetic cases, in particular for *SOD1* and *TARDBP*; therefore, the slope estimates may be imprecise. Finally, population denominators were not available for specific mutations; however, as noted above, this would have affected our age-specific incidence estimates but not the slope of the graph of log incidence against log age. It is therefore important that our findings be replicated in other populations with larger cohorts of patients to confirm our results and to determine the extent to which they can be generalized.

The identification of a reduced number of steps in patients with ALS with genetic mutations compared to those without mutations strongly supports the idea of ALS as a multistep process and represents a first clue for uncovering the pathogenic process of ALS. Similar patterns have previously been observed in studies of specific cancers in which the relevant mutations and other environmentally induced steps have been able to be identified and postulated as also being relevant to neurodegeneration.^21^ Our findings support the idea of parallels between the processes leading to carcinogenesis and those leading to ALS. The fact that only 2, 3, or 4 steps are required before disease onset in genetically mediated ALS is consistent with the concept that up to 4 of the 6 steps required for disease onset are already accounted for by inherited mutation. This idea is also consistent with the observation that penetrance corresponds to the number of steps accounted for. An alternative explanation is that the underlying etiology must differ in at least 1 step between genetic and other forms of ALS. An analysis of the influence of nongenetic risk factors should therefore also be performed to clarify their contribution to the multistep process of ALS. The relatively limited number of steps leading to ALS, compared, for example, to the complexity of the mechanisms at the base of other multifactorial diseases such as schizophrenia,^39^ provides hope for the development of an effective therapy for this devastating disease.

## Glossary

ALS: amyotrophic lateral sclerosis
CI: confidence interval
PARALS: Piemonte and Valle d'Aosta Register for ALS

## References

[1] Al-Chalabi A, Hardiman O. The epidemiology of ALS: a conspiracy of genes, environment and time. Nat Rev Neurol 2013;9:617–628. 24126629 10.1038/nrneurol.2013.203

[2] Al-Chalabi A, Pearce N. Commentary: mapping the human exposome: without it, how can we find environmental risk factors for ALS? Epidemiology 2015;26:821–823. 26414852 10.1097/EDE.0000000000000381

[3] Pearce N, Kromhout H. Occupational causes of amyotrophic lateral sclerosis: where to from here? Occup Environ Med 2017;74:83–84. 27864434 10.1136/oemed-2016-103966

[4] Ingre C, Roos PM, Piehl F, Kamel F, Fang F. Risk factors for amyotrophic lateral sclerosis. Clin Epidemiol 2015;7:181–193. 25709501 10.2147/CLEP.S37505 PMC4334292

[5] Armon C. Smoking may be considered an established risk factor for sporadic ALS. Neurology 2009;73:1693–1698. 19917993 10.1212/WNL.0b013e3181c1df48 PMC2788806

[6] Al-Chalabi A, Fang F, Hanby MF, et al. An estimate of amyotrophic lateral sclerosis heritability using twin data. J Neurol Neurosurg Psychiatry 2010;81:1324–1326. 20861059 10.1136/jnnp.2010.207464 PMC2988617

[7] Keller MF, Ferrucci L, Singleton AB, et al. Genome-wide analysis of the heritability of amyotrophic lateral sclerosis. JAMA Neurol 2014;71:1123–1134. 25023141 10.1001/jamaneurol.2014.1184 PMC4566960

[8] McLaughlin RL, Vajda A, Hardiman O. Heritability of amyotrophic lateral sclerosis: insights from disparate numbers. JAMA Neurol 2015;72:857–858. 26029874 10.1001/jamaneurol.2014.4049

[9] Al-Chalabi A, Calvo A, Chio A, et al. Analysis of amyotrophic lateral sclerosis as a multistep process: a population-based modelling study. Lancet Neurol 2014;13:1108–1113. 25300936 10.1016/S1474-4422(14)70219-4 PMC4197338

[10] Armitage P, Doll R. The age distribution of cancer and a multi-stage theory of carcinogenesis. Br J Cancer 1954;8:1–12. 13172380 10.1038/bjc.1954.1 PMC2007940

[11] Chio A, Mora G, Calvo A, et al. Epidemiology of ALS in Italy: a 10-year prospective population-based study. Neurology 2009;72:725–731. 19237701 10.1212/01.wnl.0000343008.26874.d1

[12] O'Toole O, Traynor BJ, Brennan P, et al. Epidemiology and clinical features of amyotrophic lateral sclerosis in Ireland between 1995 and 2004. J Neurol Neurosurg Psychiatry 2008;79:30–32. 17634215 10.1136/jnnp.2007.117788

[13] Chio A, Calvo A, Mazzini L, et al. Extensive genetics of ALS: a population-based study in Italy. Neurology 2012;79:1983–1989. 23100398 10.1212/WNL.0b013e3182735d36 PMC3484987

[14] Byrne S, Bede P, Elamin M, et al. Proposed criteria for familial amyotrophic lateral sclerosis. Amyotroph Lateral Scler 2011;12:157–159. 21208036 10.3109/17482968.2010.545420

[15] Bruno G, Runzo C, Cavallo-Perin P, et al. Incidence of type 1 and type 2 diabetes in adults aged 30-49 years: the population-based registry in the province of Turin, Italy. Diabetes Care 2005;28:2613–2619. 16249528 10.2337/diacare.28.11.2613

[16] Alonso A, Logroscino G, Hernan MA. Smoking and the risk of amyotrophic lateral sclerosis: a systematic review and meta-analysis. J Neurol Neurosurg Psychiatry 2010;81:1249–1252. 20639382 10.1136/jnnp.2009.180232

[17] Kioumourtzoglou MA, Rotem RS, Seals RM, Gredal O, Hansen J, Weisskopf MG. Diabetes mellitus, obesity, and diagnosis of amyotrophic lateral sclerosis: a population-based study. JAMA Neurol 2015;72:905–911. 26030836 10.1001/jamaneurol.2015.0910 PMC4975611

[18] Dupuis L, Corcia P, Fergani A, et al. Dyslipidemia is a protective factor in amyotrophic lateral sclerosis. Neurology 2008;70:1004–1009. 18199832 10.1212/01.wnl.0000285080.70324.27

[19] Sutedja NA, van der Schouw YT, Fischer K, et al. Beneficial vascular risk profile is associated with amyotrophic lateral sclerosis. J Neurol Neurosurg Psychiatry 2011;82:638–642. 21471184 10.1136/jnnp.2010.236752

[20] Ambatipudi S, Cuenin C, Hernandez-Vargas H, et al. Tobacco smoking-associated genome-wide DNA methylation changes in the EPIC study. Epigenomics 2016;8:599–618. 26864933 10.2217/epi-2016-0001

[21] Frank SA. Somatic evolutionary genomics: mutations during development cause highly variable genetic mosaicism with risk of cancer and neurodegeneration. Proc Natl Acad Sci USA 2010;107(suppl 1):1725–1730. 19805033 10.1073/pnas.0909343106 PMC2868288

[22] Lattante S, Ciura S, Rouleau GA, Kabashi E. Defining the genetic connection linking amyotrophic lateral sclerosis (ALS) with frontotemporal dementia (FTD). Trends Genet 2015;31:263–273. 25869998 10.1016/j.tig.2015.03.005

[23] Cady J, Allred P, Bali T, et al. Amyotrophic lateral sclerosis onset is influenced by the burden of rare variants in known amyotrophic lateral sclerosis genes. Ann Neurol 2015;77:100–113. 25382069 10.1002/ana.24306 PMC4293318

[24] van Blitterswijk M, van Es MA, Hennekam EA, et al. Evidence for an oligogenic basis of amyotrophic lateral sclerosis. Hum Mol Genet 2012;21:3776–3784. 22645277 10.1093/hmg/dds199

[25] Chio A, Calvo A, Moglia C, et al. ATXN2 polyQ intermediate repeats are a modifier of ALS survival. Neurology 2015;84:251–258. 25527265 10.1212/WNL.0000000000001159

[26] Diekstra FP, van Vught PW, van Rheenen W, et al. UNC13A is a modifier of survival in amyotrophic lateral sclerosis. Neurobiol Aging 2012;33:e633–e638. 10.1016/j.neurobiolaging.2011.10.029 22118904

[27] Fogh I, Lin K, Tiloca C, et al. Association of a locus in the CAMTA1 gene with survival in patients with sporadic amyotrophic lateral sclerosis. JAMA Neurol 2016;73:812–820. 27244217 10.1001/jamaneurol.2016.1114 PMC5556366

[28] Cronin S, Greenway MJ, Prehn JH, Hardiman O. Paraoxonase promoter and intronic variants modify risk of sporadic amyotrophic lateral sclerosis. J Neurol Neurosurg Psychiatry 2007;78:984–986. 17702780 10.1136/jnnp.2006.112581 PMC2117866

[29] Siu J, Perkins E, Cashman NR. Effects of sex and family history on the amyotrophic lateral sclerosis (ALS) multistep model. Amyotroph Lateral Scler 2016;17:94.

[30] Murphy NA, Arthur KC, Tienari PJ, Houlden H, Chio A, Traynor BJ. Age-related penetrance of the C9orf72 repeat expansion. Sci Rep 2017;7:2116. 28522837 10.1038/s41598-017-02364-1 PMC5437033

[31] Van Langenhove T, van der Zee J, Gijselinck I, et al. Distinct clinical characteristics of C9orf72 expansion carriers compared with GRN, MAPT, and nonmutation carriers in a Flanders-Belgian FTLD cohort. JAMA Neurol 2013;70:365–373. 23338682 10.1001/2013.jamaneurol.181

[32] Gijselinck I, Van Mossevelde S, van der Zee J, et al. The C9orf72 repeat size correlates with onset age of disease, DNA methylation and transcriptional downregulation of the promoter. Mol Psychiatry 2016;21:1112–1124. 26481318 10.1038/mp.2015.159 PMC4960451

[33] Kramer NJ, Carlomagno Y, Zhang YJ, et al. Spt4 selectively regulates the expression of C9orf72 sense and antisense mutant transcripts. Science 2016;353:708–712. 27516603 10.1126/science.aaf7791 PMC5823025

[34] Chio A, Borghero G, Pugliatti M, et al. Large proportion of amyotrophic lateral sclerosis cases in Sardinia due to a single founder mutation of the TARDBP gene. Arch Neurol 2011;68:594–598. 21220647 10.1001/archneurol.2010.352 PMC3513278

[35] Aggarwal A, Nicholson G. Age dependent penetrance of three different superoxide dismutase 1 (sod 1) mutations. Int J Neurosci 2005;115:1119–1130. 16040355 10.1080/00207450590914392

[36] Cudkowicz ME, McKenna-Yasek D, Sapp PE, et al. Epidemiology of mutations in superoxide dismutase in amyotrophic lateral sclerosis. Ann Neurol 1997;41:210–221. 9029070 10.1002/ana.410410212

[37] Andersen PM, Nilsson P, Ala-Hurula V, et al. Amyotrophic lateral sclerosis associated with homozygosity for an Asp90Ala mutation in CuZn-superoxide dismutase. Nat Genet 1995;10:61–66. 7647793 10.1038/ng0595-61

[38] Robberecht W, Aguirre T, Van den Bosch L, Tilkin P, Cassiman JJ, Matthijs G. D90A heterozygosity in the SOD1 gene is associated with familial and apparently sporadic amyotrophic lateral sclerosis. Neurology 1996;47:1336–1339. 8909456 10.1212/wnl.47.5.1336

[39] Elert E. Aetiology: searching for schizophrenia's roots. Nature 2014;508:S2–S3. 24695332 10.1038/508S2a

